# Transcriptomic Response of Mussel Gills After a *Vibrio splendidus* Infection Demonstrates Their Role in the Immune Response

**DOI:** 10.3389/fimmu.2020.615580

**Published:** 2020-12-16

**Authors:** Amaro Saco, Magalí Rey-Campos, Beatriz Novoa, Antonio Figueras

**Affiliations:** Institute of Marine Research (IIM), National Research Council (CSIC), Vigo, Spain

**Keywords:** transcriptomic, gill, mussel, immune response, RNA-seq, bath infection, transcriptome, innate immunity

## Abstract

Mussels (*Mytilus galloprovincialis*) are filter feeder bivalves that are constantly in contact with a wide range of microorganisms, some of which are potentially pathogenic. How mussels recognize and respond to pathogens has not been fully elucidated to date; therefore, we investigated the immune mechanisms that these animals employ in response to a bacterial bath infection from the surrounding water, mimicking the response that mussels mount under natural conditions. After the bath infection, mussels were able to remove the bacteria from their bodies and from the water tank. Accordingly, antibacterial activity was detected in gill extracts, demonstrating that this tissue plays a central role in removing and clearing potential pathogens. A transcriptomic study performed after a bath infection with *Vibrio splendidus* identified a total of 1,156 differentially expressed genes. The expression levels of genes contributing to a number of biological processes, such as immune response activation pathways and their regulation with cytokines, cell recognition, adhesion and apoptosis, were significantly modulated after infection, suggesting that the gills play important roles in pathogen recognition, as well as being activators and regulators of the mussel innate immune response. In addition to RNA-seq analysis, long non-coding RNAs and their neighboring genes were also analyzed and exhibited modulation after the bacterial challenge. The response of gills against bath infection was compared with the findings of a previous transcriptomic study on hemocytes responding to systemic infection, demonstrating the different and specific functions of gills. The results of this study indicate that recognition processes occur in the gill, thereby activating the effector agents of the immune response to overcome bacterial infection.

## Introduction

Mussels (*Mytilus galloprovincialis*) are distributed worldwide and exert a strong impact on community structure and ecosystems due to their interactions with other species and their effects on physical and biological conditions (1). Due to their ubiquitous distribution, mussels have been used as pollution markers for more than 40 years (2). Mussels (*Mytilus galloprovincialis* and *M. edulis*) are also the most cultured species in the European Union, being primarily produced in Galicia (NW of Spain). Mussel aquaculture production in Europe reached 493,844 tons, valued at approximately 390 million euros, in 2017 (3). Aquaculture is currently a necessary production activity to satisfy the consumption needs of the population while guaranteeing sustainable exploitation. Cultured species are susceptible to diseases that may have severe economic impacts, but unlike other bivalves, such as oysters (4), large-scale mortalities have not been reported in the field in the case of *M. galloprovincialis* (5–7). Mussels present a higher and more complex set of immune-related genes than other bivalves, which makes the molecular basis of their immune systems a topic of considerable interest (8).

Due to the filtering activity exhibited by mussels, they are constantly exposed to numerous kinds of microorganisms. It has not been elucidated to date how mussels can recognize and respond to pathogenic microorganisms. Invertebrate innate immune systems recognize pathogen-associated molecular patterns (PAMPs) and danger/damage-associated molecular patterns (DAMPs), and these mechanisms may be very effective in mussels (9–11). Most likely, mussels have developed mechanisms to avoid excessive immune reactions and to exert strong control of their inflammatory responses (12).

Molluskan hemocytes are important components of the bivalve immune system. These cells are present in all internal spaces of bivalves, circulating in the hemolymph, which bathes all tissues, and migrating into the pallial and extrapallial spaces. Different types of hemocytes have been described in mollusks based on their morphological characteristics and their roles in physiological processes (e.g., digestion and shell formation) and immune functions (e.g., phagocytosis, synthesis of immune effectors, and modulation of immune responses) (11, 13, 14). Bivalve hemocytes express a wide variety of highly variable immune effectors as antimicrobial peptides (AMPs). Some of these genes have been identified as defensins (15), myticins (16), mytilins (17), mytimycins (18), big defensins and mytimacins (19), myticusins (20), mytichitins (21), and myticalins (22).

In addition to hemocytes, the mucosal immunity functions of gills or mucus are essential in mollusk defense responses (23). Mussel gills are composed of filaments of ciliated and nonciliated epithelial cells, mucous gland cells, cuboidal respiratory epithelium, trabecular cells (which hold the filaments) and infiltrated hemocytes (24, 25). Gills are implicated in filtering the surrounding water and may be involved in mechanisms of recognition or agglutination of filtered pathogens. Pathogens able to bypass these initial barriers (either by surviving inside phagocytic cells or by directly migrating through epithelial junctions) could trigger a systemic immune response (23).

The objective of this work was to conduct, for the first time, a transcriptomic study of the gill response to an incoming bacterial bath infection. While most previously published transcriptomic approaches have focused on hemocytes, which are the effector cells of the immune response against systemic infections, with the experimental design of this study, it may be possible to demonstrate how mussels recognize potential infections from the surrounding water. *Vibrio splendidus* was chosen as the pathogen because it has been shown that under experimental conditions, this species could be pathogenic for mussels (5, 26). The tissue distribution of the bacterial load in wild mussels and bath-infected mussels was also studied, as well as the antibacterial activity of those tissues.

## Materials and Methods

### Animals

Adult mussels (*Mytilus galloprovincialis*), of 9 cm shell-length, were obtained in February from a commercial mussel farm (Vigo, Galicia, Spain) and maintained in open-circuit filtered seawater (FSW) tanks at 15°C with aeration. The mussels were fed daily with *Phaeodactylum tricornutum* and *Isocrhysis galbana*. The mussels were used for the experiments after at least one week of acclimatization.

Wild mussels (5 cm shell-length) sampled in February from intertidal rock substrates in Alcabre (Ría de Vigo, Vigo, Galicia, Spain) were used for the study of the basal bacterial load in mussels in the natural environment.

### Bacterial Infection

*Vibrio splendidus* (reference strain LGP32) was cultured in tryptic soy agar (TSA) plates at 22°C for 24 h before use. Bacterial suspensions were prepared in FSW before infection. Serial dilutions were made to calculate the number of colony forming units (CFU)/ml 24 h after inoculation.

Bath infections were performed (at 09:00 a.m.) by treating mussels with a concentration of 10^8^ CFU/ml *V. splendidus* in tank water. Control mussels were maintained in another tank with filtered seawater (FSW). Samples from control and infected mussels were taken (09:00 a.m.) at days 1, 3, 6, and 8 after *V. splendidus* infection for the determination of bacterial load in different tissues. Additionally, gills from infected and control mussels were collected for RNA extraction.

### Bacterial Load

Hemocytes, hemolymph serum, intervalvar liquid, mucus, gills and mantle were taken from mussels sampled from the wild population to study their basal bacterial load under natural conditions. The same procedure was performed with five control and five infected mussels at days 1, 3, 6, and 8 after the bath infection.

Hemolymph samples (1 ml) were withdrawn from the adductor muscle of each mussel with a 0.5-mm-diameter (25 G) disposable needle through a notch on the shell. Hemolymph was centrifuged at 4°C at 1 g for 20 min. Serum was recovered, and the hemocytes pellet was suspended in FSW (1 ml). After the valve opening, the intervalvar liquid and mucus were collected. Finally, gills and mantle were sampled, weighed and homogenized in FSW (200 µl).

Serial dilutions were made of all samples and plated in triplicate in TSA + 1.5% NaCl culture plates. Colony counts were performed 24 h later, and the bacterial concentration was calculated in CFU/ml. In tissue samples (gill and mantle), bacterial concentration was normalized with respect to the weight (in mg) of each sample.

### Antibacterial Activity

Hemolymph, hemocytes, serum, gill and mucus samples were obtained from non-stimulated mussels (as previously described). Samples were homogenized by being passed through a syringe and needle to obtain a lysate. The samples were all prepared in sterile filtered seawater (FSW) supplemented with the same 1:1 proportion of TSB liquid culture medium and filtered through 0.22-μm diameter filters. The antibacterial activity of the samples was tested with 1x10^6^ CFUs/ml *Vibrio splendidus* in a 1:10 ratio in triplicate on a 96-well plate. Bacterial and extract controls were also employed.

Bacterial growth was determined by optical density at 620 nm after 10 h, and the inhibition rate over the bacterial growth was calculated for each tissue extract by relativizing the optical density values of bacterial controls vs. those of treatments.

### RNA Isolation, cDNA Production, Real-Time PCR, and Illumina Sequencing

RNA extraction was performed on three controls and three infected gill samples from each sampled day using the Maxwell 16 LEV simplyRNA kit (Promega) following the manufacturer’s protocol. The concentration of RNA was measured using a NanoDrop ND1000 spectrophotometer (NanoDrop Technologies).

cDNA was synthesized by reverse transcription from 300 ng of total RNA of each sample using the NZY First-Strand cDNA Synthesis Kit (Nzytech) according to the manufacturer’s protocol. *Vibrio splendidus* was detected by real-time PCR with specific primers (forward: ATCATGGCTCAGATTGAACG; reverse: CAATGGTTATCCCCCACATC) and TaqMan PCR probe (FAM-CCCATTAACGCACCCGAAGGATTG-BHQ1) following the method described by *Saulnier* et al. (27).

Three control and infected RNA gill samples from day 1 were used for sequencing using NGS Illumina HiSeq™ 4500 technology at Macrogen Inc. Korea (Seoul, Republic of Korea).

The raw reads were deposited in the SRA-NCBI database with the following accession numbers: SRR11996464, SRR11996686 and SRR11996723 for control samples and SRR11996734, SRR11996735 and SRR11996743 for infected samples, all of which are accessible *via* the Bioproject PRJNA638821.

### Bioinformatics and RNA-Seq

Transcriptomic analysis was performed on the six sequenced gill samples from six individuals (three controls and three infected individuals). CLC Genomics Workbench, v.12. software package (CLC Bio, Qiagen) was used to filter, assemble and perform the RNA-seq and the statistical analyses of individual mussels.

Forward and reverse reads generated by Illumina sequencing of each sample were paired using CLC Genomics Workbench, v.12. (CLC Bio, Qiagen). The number of raw reads was highly similar for each sequenced mussel (**Table 1**). For each individual transcriptome, raw reads were trimmed to remove Illumina HiSeq™ 4500 adapter sequences and short and low-quality sequences (quality score limit 0.01 = PHRED 20). Next, a reference transcriptome for the six mussel gill libraries was *de novo* assembled with an overlap criterion of 70% and a similarity of 90% to exclude paralogous sequence variants. The assembly settings were a mismatch cost = 2, deletion cost = 3, insert cost = 3, and a minimum contig length = 200 base pairs.

**Table 1:** Summary of the bioinformatic results of the transcriptomic analysis.

Sequenced reads	Raw reads	Trimmed reads
C1	24,096,934	24,096,915
C2	21,582,336	21,582,332
C3	19,815,334	19,815,330
I1	17,170,648	17,170,647
I2	17,690,744	17,697,042
I3	25,179,632	25,179,628
Mean	20,923,654	20,923,649
***De novo* assembly**		
Contigs	162,167	
Average contig length	550	
N50	653	
**Blast annotation**		
UniProt/Swiss-Prot	35,339 annotated contigs (22%)	
Mollusk database	88,133 annotated contigs (54%)	
**RNA-seq differential expression analysis**		
Differential expressed genes (DEGs)	1,156 contigs	
Annotated DEGs	401 contigs	
**GO enrichment analysis**		
Up-modulated	63 biological processes	
Downregulated	41 biological processes	
**Long non-coding RNA analysis**		
Non-annotated contigs	126,828 contigs	
Potential lncRNAs	6,021 contigs	
lncRNAs mapped on the genome	2,130 (35%)	
Modulated lncRNAs (RNA-seq)	413	
Modulated and mapped lncRNAs	78	

RNA-seq analysis was performed by mapping the paired trimmed reads of each individual to the assembled reference global transcriptome, considering maximum hits per read = 10, length fraction = 0.8 and similarity fraction = 0.8. Expression values were set as transcripts per million (TPM). A differential expression analysis test (Robinson and Smyth’s Exact Test, which assumes a negative binomial distribution of the data and considers the overdispersion caused by biological variability) was used to compare expression levels in each sample and to identify the differentially expressed genes (DEGs). Transcripts with absolute fold change (FC) values > 2 and FDR (Benjamini and Hochberg’s False Discovery Rate) < 0.05 were selected.

### BLAST Annotation, GO Assignment, and Enrichment Analysis

Contigs obtained in the *de novo* assembly were annotated using OmicsBox software (BioBam). Annotation was performed with BLASTx against UniProt/Swissprot and BLASTn against an in-house built database with all the mollusk sequences present in the NCBI (National Center for Biotechnology Information). For both BLAST analyses, a threshold value of 1e-3 was set. Also, gene ontology (GO) terms were assigned to the annotated contigs using the same software. Enrichment analyses of the up- and downregulated genes (DEGs) were also conducted using the assembled global transcriptome as a reference. Fisher’s exact test was performed using a p-value cut-off of 0.01.

### Long Non-Coding RNA Mining and Differential Expression Analysis

Using CLC Genomics Workbench, non-annotated contigs were retrieved from the *de novo* assembly, and reads were mapped to those contigs to retain only those with an average coverage > 50 and a length of at least 200 bp. For the selection of putative long non-coding RNAs (lncRNAs), contigs were selected after discarding every possible coding sequence using three filters: potential open reading frame (ORF) prediction, BLASTx against the mussel genome peptide database (28) and coding potential prediction with the Coding Potential Assessment Tool CPAT (29).

RNA-seq analyses of the putative lncRNAs were conducted using the same methodology used for the differential gene expression analysis.

### Genome Mapping and Identification of lncRNA Neighboring Coding Genes

Putative lncRNA contigs were mapped to the scaffolds of the mussel genome (28) with length fraction = 0.75 and similarity fraction = 0.98. The coding genes located up to 10 kb upstream and downstream of the modulated lncRNAs were identified and selected for GO enrichment analyses with OmicsBox software (Fisher’s exact test, FDR < 0.05). Neighboring gene expression was retrieved from the gill transcriptome, and the correlation with the expression of their corresponding modulated lncRNAs was studied by calculating Pearson’s correlation coefficients with R software. Heatmaps were made using ClustVis (30).

### Gill Transcriptomic Response Comparison With Hemocyte Transcriptomic Response

Venn diagram analyses were performed using the Venny 2.1 tool (31) to compare DEGs of mussels infected using two separate experimental approaches: the current gill transcriptomic study against a bath infection and a transcriptomic analysis of hemocytes from mussels infected by injection with the same bacteria retrieved from a previous work and accessible *via* Bioproject PRJNA466718 (32). Assembly, annotation and differential expression analysis were performed following the same pipeline explained above such that the two approaches were comparable. DEGs for this transcriptome were obtained and retrieved for the comparison between three infected mussels injected with *Vibrio splendidus* in the adductor muscle and three control mussels injected with FSW (32), both at 24 h after injection, to make them comparable to the current work. Annotated DEGs from both studies were compared, and common and exclusively modulated genes were selected. GO enrichment analyses were performed for those common/exclusively annotated DEGs using DAVID Bioinformatics Resources 6.8. (Laboratory of Human Retrovirology and Immunoinformatics, LHRI) (33, 34).

### Quantitative PCR (qPCR) Validation of the RNA-Seq Analysis

Validation of the RNA-seq analysis was carried out using the same RNA samples from the gill transcriptome. qPCRs of selected genes (**Supplementary File 1**) were performed in a 7300 Real Time PCR System (Applied Biosystems). Cycling conditions were 95°C for 10 min followed by 40 cycles of 95°C for 15 s and 60°C for 30 s. Reactions were performed with technical duplicates, and the relative expression was normalized using 18S as a housekeeping gene following the Pfaffl method (35). Pearson’s correlation analysis between quantitative PCR (qPCR) and transcriptome data was performed and calculated with R software (**Supplementary File 1**).

## Results

### Bacterial Load in Infected and Uninfected Mussels

**Figure 1:**
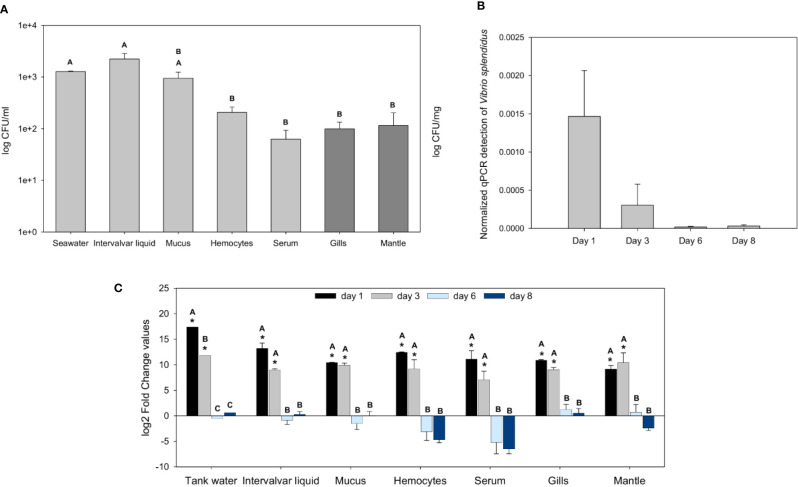
Analysis of the bacterial load in naïve wild mussels and in mussels submitted to the experimental bath infection. **(A)** Constitutive bacterial load distribution in freshly sampled wild rock mussels (N = 17). The bacterial concentration (mean ± SEM) is represented in colony forming units (CFUs)/ml for every sample except tissue samples, which were normalized to their weight (CFUs/mg). Nonparametric Tamhane’s T2 *post hoc* tests were used to search for significant differences among tissues, displayed with different letters (p-value < 0.05). **(B)** Normalized *Vibrio splendidus* detection by quantitative PCR (qPCR) on infected mussels gill samples (N = 3) 1, 3, 6, and 8 days after the bath infection (ANOVA; p-value < 0.05). **(C)** Fold change values (mean ± SEM; N = 5) of the bacterial load differences (CFUs) between infected and control mussel samples after bath infection with *Vibrio splendidus*. Statistical differences between groups were studied by applying a generalized linear model (GLM) considering time, sample and infection status as factors with Bonferroni *post hoc* tests to study significant differences (p-value < 0.05). Significant differences are shown with asterisks for the comparisons between infected and control bacterial load values and with letters for the comparisons between days.

First, the bacterial abundance in wild mussels from the natural environment was evaluated. The intervalvar liquid was the mussel sample that showed the highest amount of bacterial load, higher than that of seawater. In the other samples and tissues, the bacterial abundance was lower: in mucus, there was a significant bacterial concentration, but it decreased significantly in the hemolymph, gills and mantle (**Figure 1A**).

After bath infection with *V. splendidus*, the bacterial load increased significantly in the various samples. The specific detection of *V. splendidus* in gills by qPCR showed high levels of *V. splendidus* after infection at days 1 and 3 that decreased at days 6 and 8 with complete clearance of the pathogen (**Figure 1B**). At days 1 and 3 post-infection, every mussel sample and the tank water showed bacterial loads considerably higher than those of the controls (**Figure 1C**). Six and 8 days after the infection, the bacterial levels of the infected animals were not significantly different from those of the controls, suggesting that the bacteria were cleared and returned to basal values similar to those of the uninfected control animals.

### Antibacterial Activity of Different Mussel-Derived Extracts

**Figure 2:**
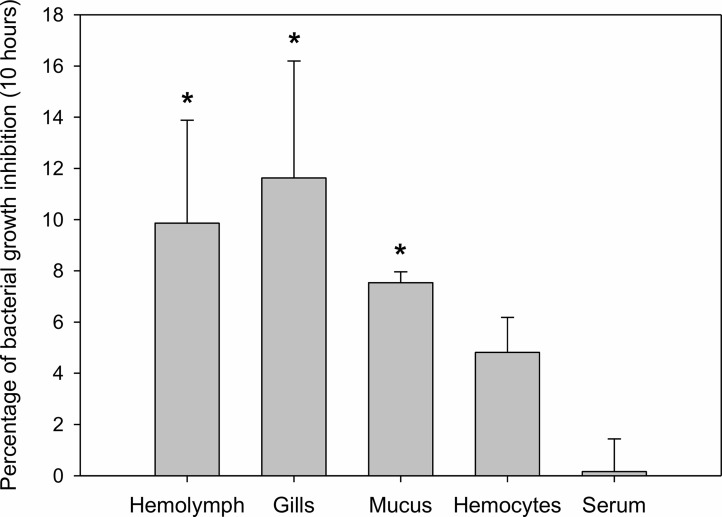
Antibacterial activity of different mussel extracts. Antibacterial activity is represented as percentages of inhibition over bacterial growth at a final time of 10 h. Significant inhibition is displayed with asterisks for those treatments in which the bacterial growth was significantly reduced by the extracts compared to the control bacterial growth (t-test, p-value < 0.05).

Mussel homogenates showed different levels of antibacterial activity (**Figure 2**). Gill homogenates were the most effective, reaching an inhibition of 11% of the bacterial concentration. Mucus, hemolymph and hemocytes homogenates also showed some antibacterial activity (7%, 10%, 5% inhibition), while serum (mussel hemolymph without hemocytes) exhibited less antibacterial activity.

### Mussel Gill Transcriptomic Response After a Bacterial Bath Infection

Gill tissue was subsequently selected to perform RNA-seq analysis after bath infection. The 6 sequenced gill samples had a similar number of reads with an average of 20.9 million raw reads (**Table 1**). The common assembly of the reference transcriptome resulted in 162,167 contigs with an average length of 550 bp. OmicsBox software was used to successfully identify and annotate 22% of contigs by BLASTx against UniProt/Swiss-Prot. Moreover, a local BLASTn using CLC Workbench enabled us to annotate 54% of the *de novo* assembly contigs using an in-house designed database with all the nucleotide sequences available in NCBI for mollusks.

**Figure 3:**
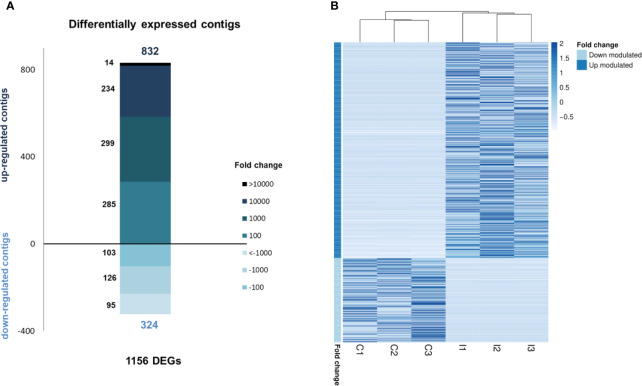
RNA-seq analysis of the transcriptomic response in gills against bath infection. **(A)** Distribution of the fold change values of the differentially expressed genes (DEGs). **(B)** Expression levels (TPMs) of all DEGs in the control and infected samples.

RNA-seq analysis demonstrated that 1,156 genes were modulated with infection, i.e., 832 upregulated and 324 downregulated, suggesting a clear activation of gene transcription in gills caused by bath infection (**Figure 3A**). The expression values (TPMs) of those modulated genes are represented in a heatmap (**Figure 3B**).

**Figure 4:**
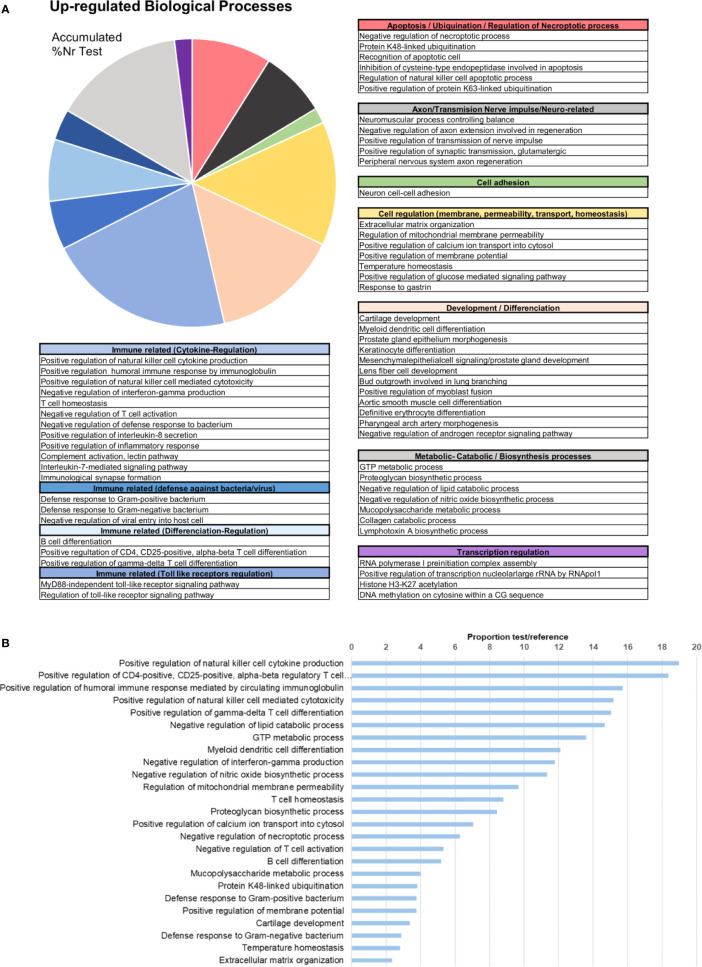
Enrichment analysis of the upregulated differentially expressed genes (DEGs). **(A)** Biological processes classified in accordance with their biological functions and represented with their %Nr Test values (proportion of DEGs represented for each biological process among the total number of upregulated genes)**. (B)** Enrichment analysis showing the most represented biological processes (proportion between the test and the reference set).

In terms of GO information, the upregulated biological processes after bath infection were clustered into several categories depending on their function (**Figure 4A**). The fact that many of these processes were related to the recognition of pathogens, immune response activation pathways and cytokine production confirms that a recognition action toward infection and subsequent activation of the immune response takes place in gills. The five most represented upregulated processes were all immune-related: “positive regulation of natural killer cell cytokine production”, “positive regulation of CD4-positive, CD25-positive, alpha-beta regulatory T cell differentiation”, “positive regulation of humoral immune response mediated by circulating immunoglobulin”, “positive regulation of natural killer cell mediated cytotoxicity” and “positive regulation of gamma-delta T cell differentiation” (**Figure 4B**). The fact that some of these terms correspond to vertebrate biology features is a typical effect of using Gene Ontology with non-model species. Other biological processes of interest were also identified, such as those related to apoptosis regulation, cell and membrane regulation or adhesion and transmission functions.

**Figure 5:**
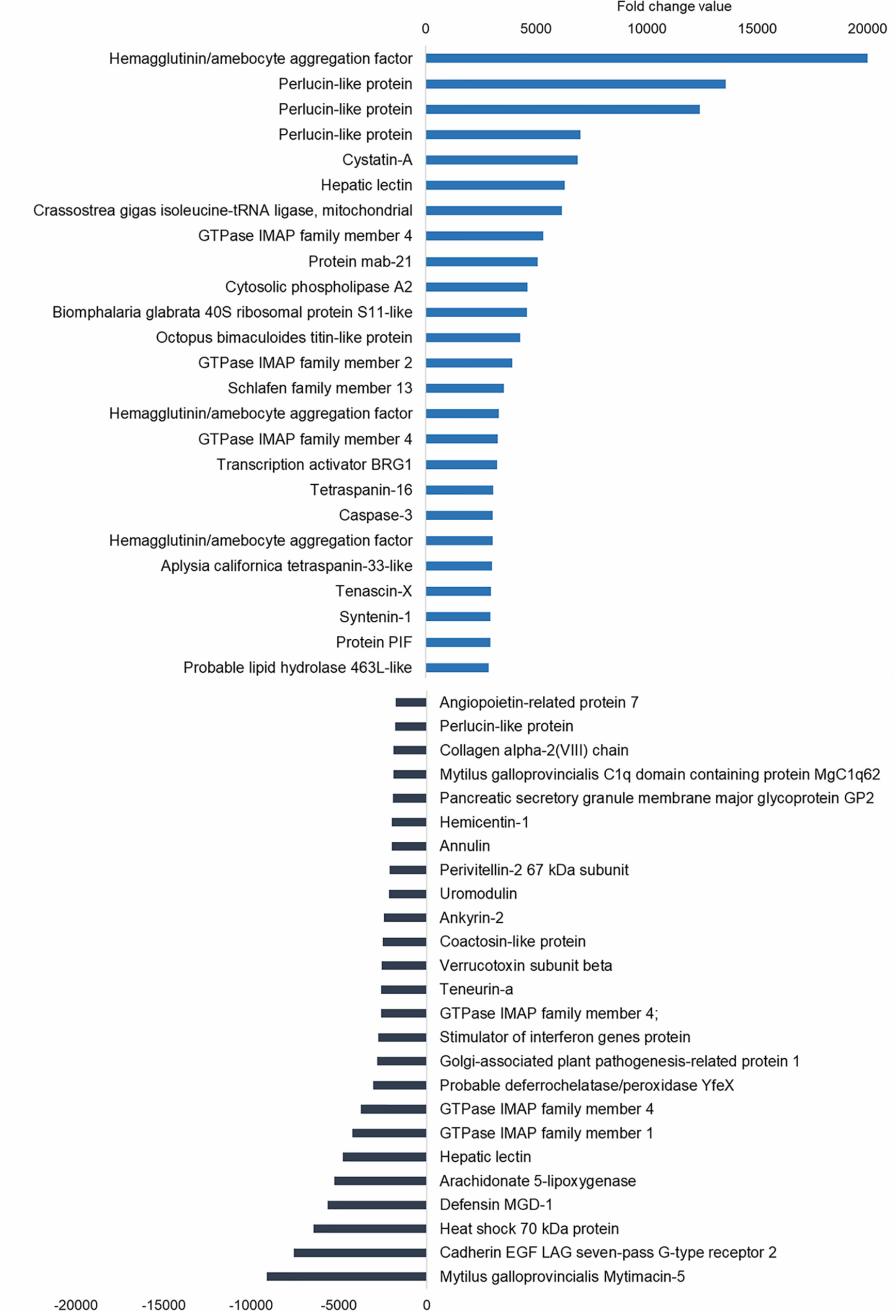
Top 25 upregulated and downregulated differentially expressed genes (DEGs) associated with bath infection in gills. Most upregulated and most downregulated genes are displayed with their fold change values.

The upregulated differentially expressed genes (DEGs) included genes with recognition and immune roles. The haemagglutinin/amoebocyte aggregation factor, cytosolic phospholipase A2, syntenin-1, tetraspanins, cystatin-A, tenascin-X and other immune cellular receptors and adhesion factors were among the most strongly upregulated genes (**Figure 5**). More immune signaling-related genes were exclusively upregulated in gills as the cause of immune-related biological processes: genes from interleukin 17 (IL-17) and Toll-like receptor (TLR) pathways, fibrinogen-related proteins (FREPs) and ficolins, among others (**Table 2**). TLRs were particularly abundant in the transcriptome with 351 contigs. The expression values of these Toll-like receptors are shown in **Supplementary File 2**.

**Table 2:** Modulated genes responsible for the up- and downregulated immune-related biological processes in the enrichment analyses.

Upregulated DEGs	FC	Downregulated DEGs	FC
Cytosolic phospholipase A2	4,601.4	GTPase IMAP family member 1	−4,247.8
Transcription activator BRG1	3,245.3	Stimulator of interferon genes protein	−2,753.4
Fibrinogen C domain-containing protein 1	1,637.1	GTPase IMAP family member 5	−2,609.5
GTPase IMAP family member 4	1,314.1	Uromodulin	−1,644.3
Adhesion G protein-coupled receptor B1	1,192.8	GTPase IMAP family member 8	−1,371.2
Baculoviral IAP repeat-containing protein 7-B	1,166.2	Complement C1q-like protein 4	−1,302.3
GTPase IMAP family member 3	1,146.3	Serum amyloid A protein	−629.5
Immune-associated nucleotide-binding prot.4	1,127.3	Immune-associated nucleotide-binding prot.7	−246.8
Galectin-3-binding protein	893.3	Caspase-2	−223
Death-associated inhibitor of apoptosis 2	762.6	Ankyrin 1	−192.4
Crustapain	500.1	Pancreatic secretory granule Mb. Prot. GP2	−184.7
GTPase IMAP family member 7	405.1	GTPase IMAP family member 7	−179.9
Death-associated inhibitor of apoptosis 1	200.2	Caspase-3	−112.2
Toll-like receptor 6	178.1	Interferon-induced helicase C domain- prot.1	−110.8
Fibrinogen-like protein A	119.2	GTPase IMAP family member 4	−57.5
Baculoviral IAP repeat-containing protein 7-A	88.3	Neurogenic locus notch homolog protein 2	−55.5
Ficolin-1	61.3	Transcription intermediary factor 1-beta	−40.2
Perivitellin-2 67 kDa subunit	42.2	Filamin-C	−30.8
Interleukin 17-like protein	40.5	Hepatocyte growth factor receptor	−24.9
Ubiquitin-conjugating enzyme E2 8	35.3		
Tenascin-R	29.7		
Toll-like receptor 4	26.8		
SVEP1 Sushi, nidogen. EGF- protein 1	22.9		
Toll-like receptor 5	12.9		

Redundant contigs for each group were represented by the one with the largest fold change value (FC).

**Figure 6:**
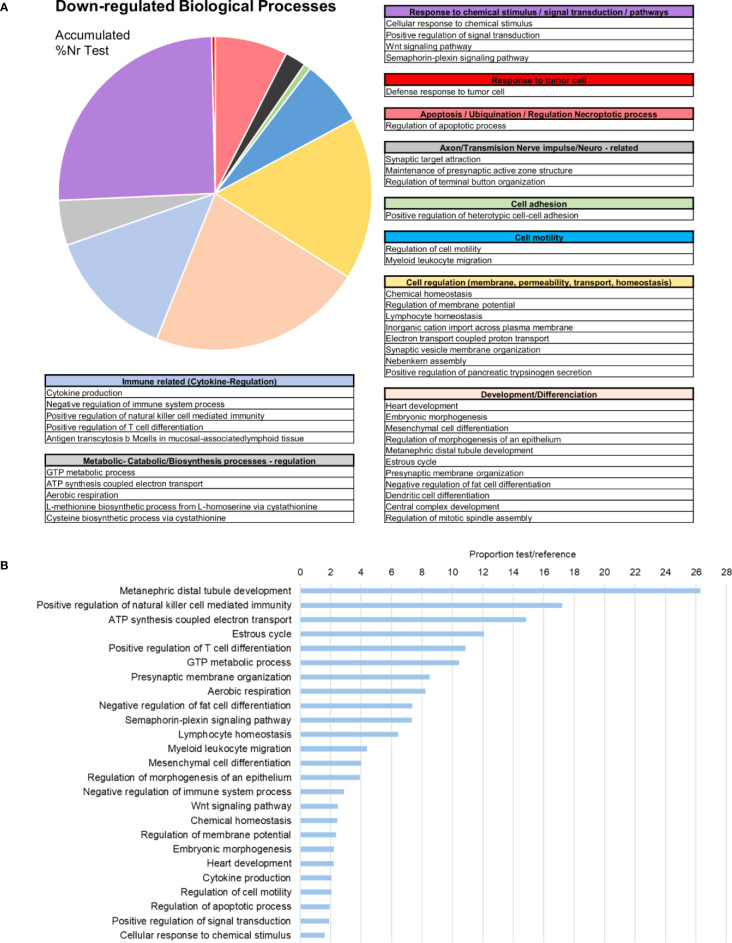
Enrichment analysis of the downregulated differentially expressed genes (DEGs). **(A)** Biological processes classified in accordance with their biological functions and represented with their %Nr Test values (proportion of DEGs represented for each biological process divided by the total number of downregulated genes). **(B)** Enrichment analysis showing the most represented biological processes (proportion between the test and the reference set).

Subsequent analysis focused on the downregulated biological processes (**Figure 6A**) and determined that the weight of immune terms is less important. The most represented biological processes were “metanephric distal tubule development”, “positive regulation of natural killer cell mediated immunity”, “ATP synthesis coupled electron transport”, “oestrous cycle” related to hormone regulation in mussels and “positive regulation of T cell differentiation” (**Figure 6B**). Mytimacin and defensin, the only two modulated antimicrobial peptide (AMP) genes, were determined to be among the most downregulated DEGs, as displayed in **Figure 5**. Genes related to the metabolism of arachidonic acid, such as the arachidonate 5-lipoxygenase and the coactosin-like protein, the cadherin EGF LAG seven-pass G-type receptor 2, the immunosuppressive uromodulin homolog or Tamm-Horsfall glycoprotein (THP), the Golgi-Associated Plant Pathogenesis-Related Protein GAPR-1 and Teneurin-a synaptic adhesion molecule, were also among the most downregulated genes (**Figure 5**).

### lncRNA Modulation After Infection

**Figure 7:**
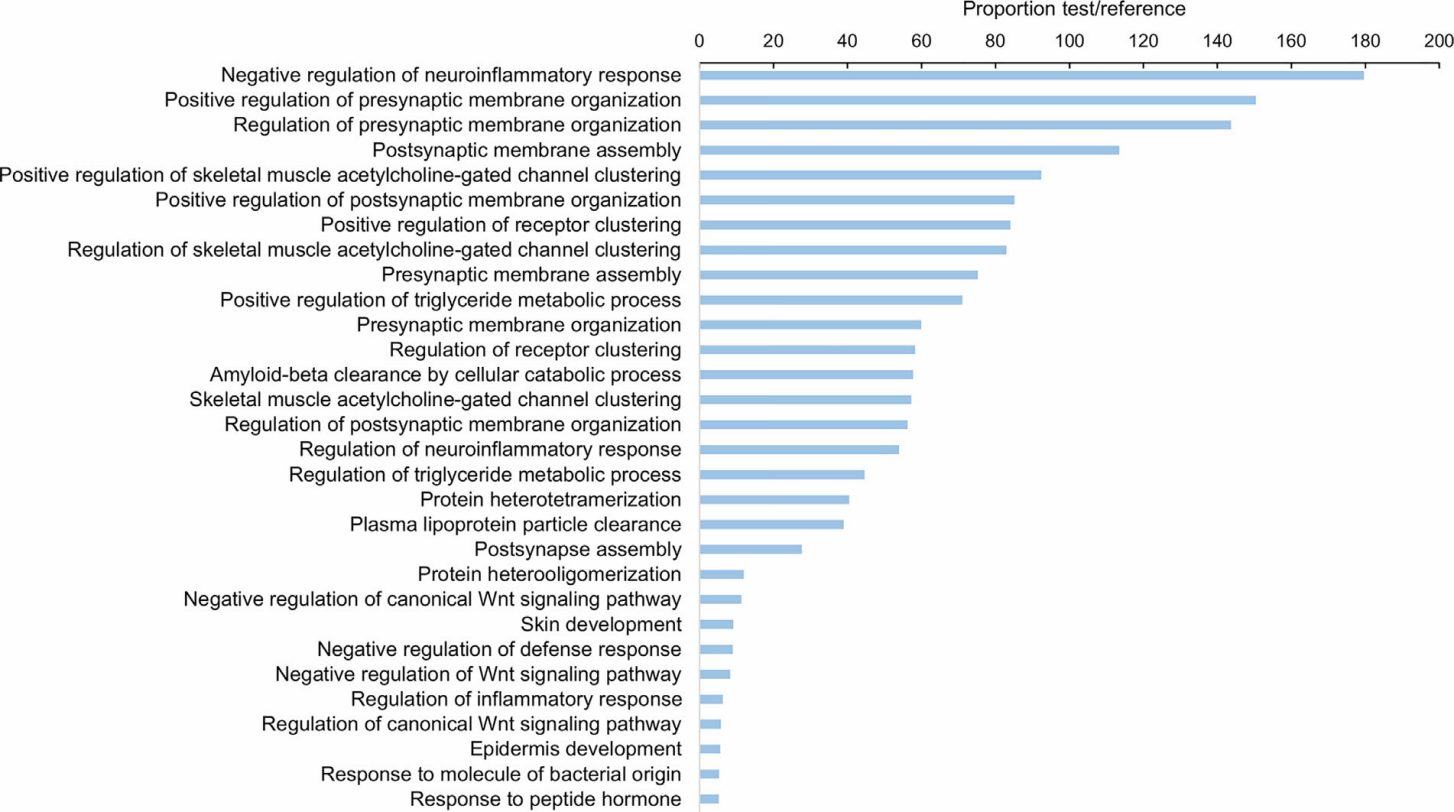
Gene ontology (GO) enrichment analysis (biological processes) of the neighboring coding genes of the differentially expressed lncRNAs in gill after bacterial bath infection. Only the 30 most significant terms were represented.

**Figure 8:**
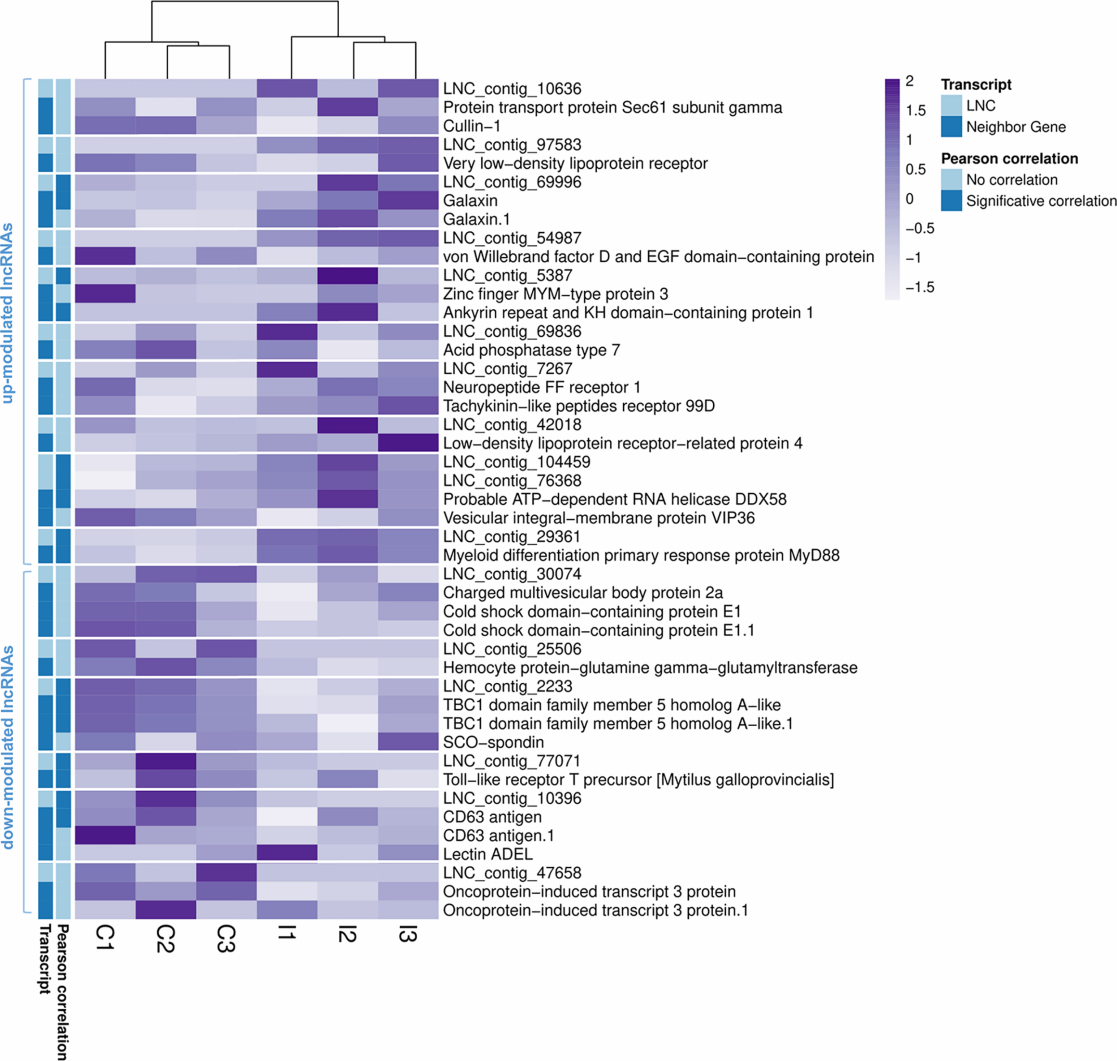
Correlation between differentially modulated lncRNAs after infection and their annotated neighboring genes. Heatmap representing the transcripts per million (TPM) values of the modulated lncRNAs and the annotated neighboring genes for each of them. Transcripts with significant Pearson correlations are also indicated.

As **Table 1** shows, 413 lncRNAs were significantly modulated by bacterial bath infection. The enrichment analysis performed on the coding genes adjacent to modulated lncRNAs (that potentially are regulated by the lncRNAs) showed a relation with the immune response. “Negative regulation of neuroinflammatory response”, “negative regulation of defense response”, “regulation of inflammatory response”, and “response to molecule of bacterial origin” were some examples of the enriched immune biological processes (**Figure 7**). The expression of modulated lncRNAs and their annotated neighboring genes was represented, and many of those genes related to recognition and immune response had significant expression correlation with their associated lncRNAs (**Figure 8**). Significant correlations were established between modulated lncRNAs and such genes as galaxin (DEG), an ankyrin protein, a Toll-like receptor precursor, MyD88, a CD63 antigen coding gene and the ATP-dependent RNA helicase DDX58, all of which are implicated in the modulated immune response against infection in gills (**Table 3**).

**Table 3:** Correlation values (Pearson’s correlation coefficient) between the lncRNAs and their significantly related coding genes.

lncRNA	Neighbor Gene	Pearson Correlation	LNC Modulation
LNC_contig_69996	Galaxin (contig 19523)	0.3109	Upregulated
	Galaxin (contig 28552)	0.9174*	
LNC_contig_5387	Zinc finger MYM-type protein 3	0.0281	Upregulated
	Ankyrin repeat and KH domain-containing protein 1	0.9478**	
LNC_contig_104459	LNC_contig_76368	0.9936***	Upregulated
	Probable ATP-dependent RNA helicase DDX58	0.9876***	
	Vesicular integral-membrane protein VIP36	−0.6281	
LNC_contig_76368	LNC_contig_104459	0.9936***	
	Probable ATP-dependent RNA helicase DDX58	0.992***	
	Vesicular integral-membrane protein VIP36	−0.6924	
LNC_contig_29361	Myeloid differentiation primary response protein MyD88	0.9897***	Upregulated
LNC_contig_10396	CD63 antigen (contig 18217)	0.828*	Downregulated
	CD63 antigen (contig 6650)	−0.1165	
	Lectin ADEL	−0.3506	
LNC_contig_2233	TBC1 domain family member 5 homolog A-like (contig 481)	0.987***	Downregulated
	TBC1 domain family member 5 homolog A-like (contig 1792)	0.9819**	
	SCO-spondin	0.066	
LNC_contig_77071	Toll-like receptor T precursor [Mytilus galloprovincialis]	0.8609*	Downregulated

Significant correlations are indicated with ***(p < 0.0005), **(0.0005 < p < 0.005), and *(0.005 < p < 0.05), and no correlated genes are shaded in gray.

### Transcriptomic Comparison Between a Systemic Infection (Hemocytes) and a Bath Infection (Gills)

**Figure 9:**
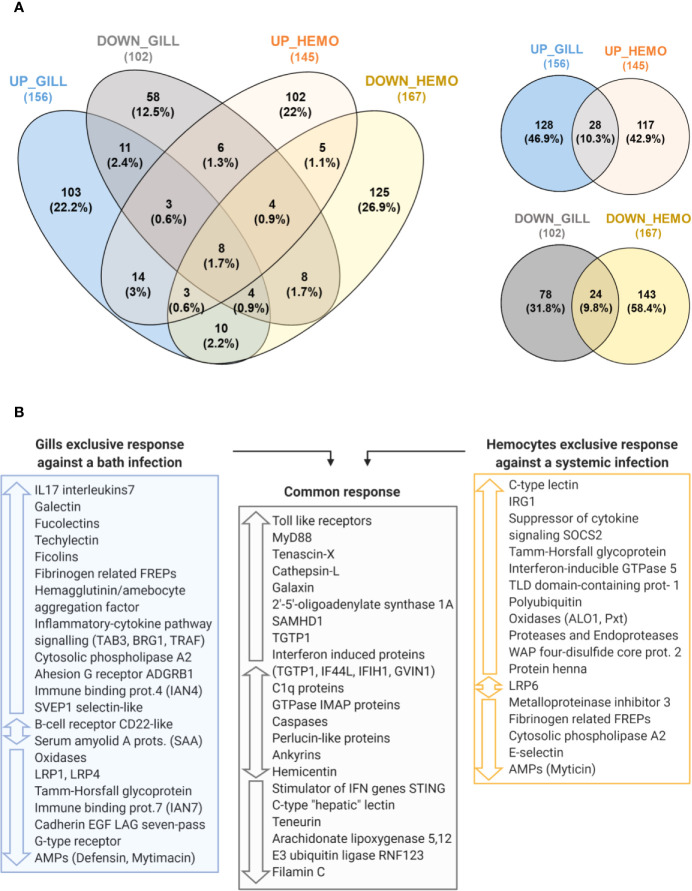
Comparison between the transcriptomic responses in gill against a bath infection and in hemocytes against a systemic infection. **(A)** Venn diagrams representing the number and percentages of shared and unique annotated differentially expressed genes (DEGs) for the comparisons between the modulated response in gill and hemocytes. **(B)** The main immune-related DEGs from each transcriptomic response are shown and classified according to whether they are exclusive to one transcriptomic response if they were commonly found in both of them.

The transcriptomic response of mussel gills to a bath infection and the response of hemocytes against an injected systemic infection (32) were notably different in terms of gene expression (**Figure 9A**). From a total of 156 nonredundant, annotated and upregulated terms in gills, 128 were exclusive when compared with the upregulated response in hemocytes. Furthermore, enrichment analyses performed in the gill-exclusive Venn diagram sector revealed the same processes related to immune response and activation, such as “Toll-like receptor signaling pathway”, “regulation of cytokine secretion”, “pattern recognition receptor signaling pathway” and “cytokine secretion” (**Supplementary File 3**). Enrichment analyses also showed biological processes, such as “defense response to virus”, “response to virus” and “defense response to other organism”, to be common to both transcriptomes due to such genes as the interferon-induced proteins IFIH1 and IF44L and the immune signaling mediator SAMHD1 (**Supplementary File 3**). As depicted in **Figure 9B**, important immune-related genes were modulated in both transcriptomes: TLRs and MyD88, the stimulator of interferon genes STING, as well as different interferon-induced proteins, recognition terms, such as C1q and perlucin-like proteins, immune signaling mediators, such as the E3 ubiquitin-protein ligase RNF123, and effector proteins, such as TGTP1 and cathepsins.

**Figure 10:**
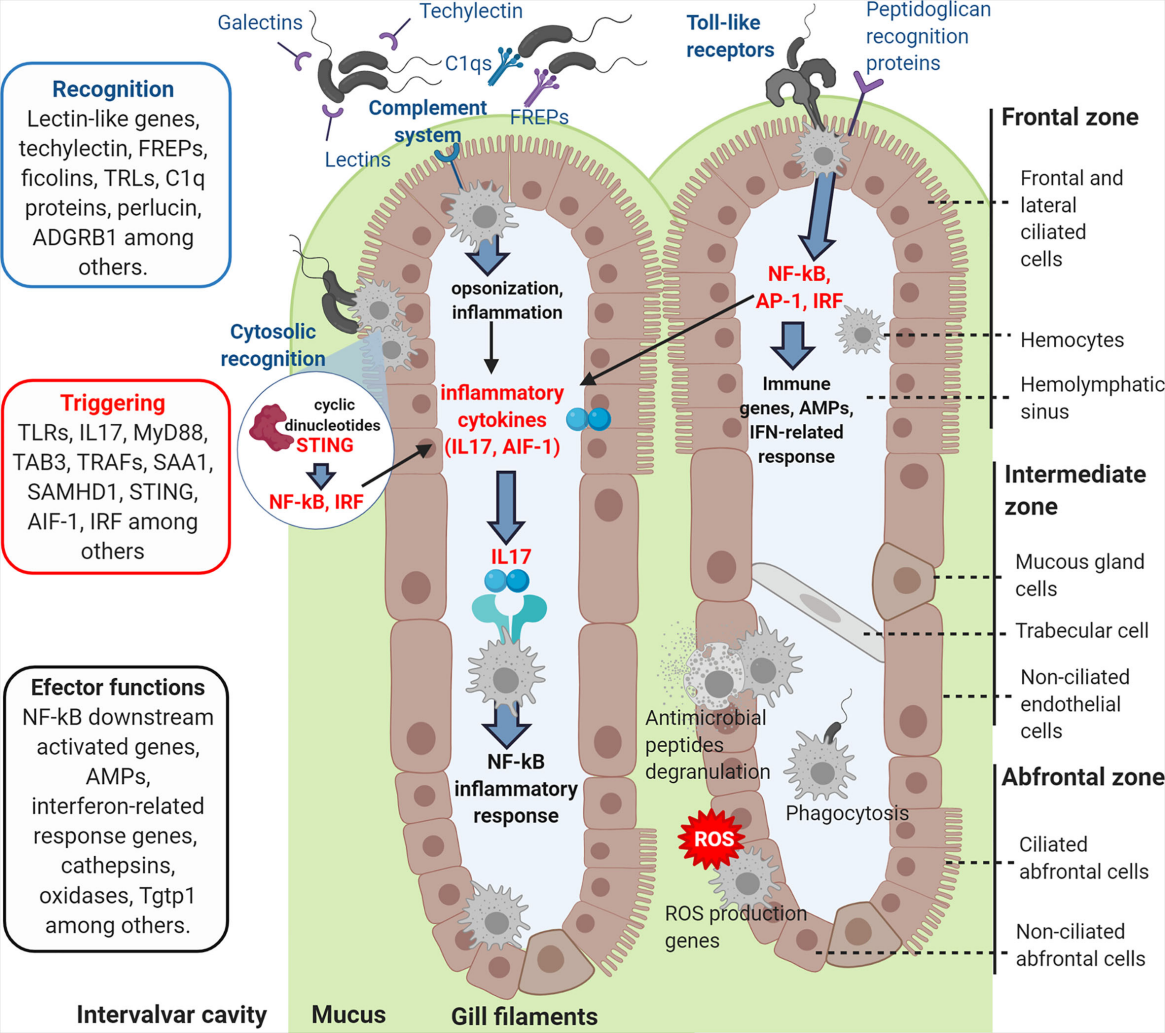
Graphic representation of how the recognition and signaling immune genes exclusively modulated in gills could recognize the incoming bacterial infection, triggering the immune response and activating the effector functions of the hemocytes.

However, gills presented an important exclusive response with DEGs absent in the transcriptome of hemocytes (**Figure 9B**), which were primarily related to immune recognition functions (FREPs, lectin-like recognition proteins, adhesion proteins) and pro-inflammatory signaling (IL17s, TRAF, TAB3, BRG1, LRP1, among others) to trigger the immune response, as represented in **Figure 10**. In hemocytes, exclusive DEGs were more heavily involved in immune effector functions, such as IRG1 and various immune oxidases and proteases. Terms more closely related to recognition, such as FREPs, were downregulated in hemocytes, while a suppressor of cytokine signaling was exclusively upregulated (**Figure 9B**).

## Discussion

Mussels are filtering bivalves that are constantly exposed to a high number of microorganisms, some of which are potentially pathogenic. The lack of mortality events observed among mussels in the natural environment suggests that their immune systems possess adequate recognition and clearance mechanisms to defend themselves against potential pathogenic microbes (6, 36, 37). In our work, the bacterial concentration in natural conditions of wild mussels was higher in the intervalvar liquid than in the seawater, as it had been reported before due to their bioaccumulative capacities (38). A decrease in the bacterial load was observed in the internal tissues, probably due to the action of external barriers and immune defenses. After mimicking a natural bath infection, infected mussels were able to remove the excess of bacterial load caused by the *Vibrio splendidus* infection in about 6 days, returning to the bacterial distribution similar to the basal levels studied before in the naïve mussels. This bacterial clearance capacity could be related to the ability of specifically recognition of the pathogenic bacteria. In the case of *Vibrio aestuarianus*, for example, the mussel extrapallial protein (MgEP), which is an opsonine present in the serum, enables the specific binding of this bacteria to membrane receptors of hemocytes, triggering bactericidal mechanisms (39). Similar mechanisms may also be utilized to prevent invasion by *Vibrio splendidus*, a pathogenic bacterium that has been reported to induce a notable degree of infection to *Mytilus galloprovincialis* (26). The mussel microbiota is less dominated by Vibrio species than other bivalve species with higher mortalities, such as the oyster *Crassostrea gigas*, which appears to be less effective in clearing these bacterial infections (40).

Gills are among the first mussel barriers that putative pathogenic bacteria from the surrounding water would confront. In this experiment, the transcriptomic response of gills was studied to determine its implication in mucosal immunity mechanisms against infection. In fact, we were able to demonstrate antibacterial activity in *Mytilus galloprovincialis* gills, in a similar way as it has been previously reported for other tissues in other mollusk species (41–43). Gills showed higher antibacterial activity than other tissues. The transcriptomic analysis also revealed that gills could be responsible of recognizing the bacterial infection when filtering the surrounding water, triggering defense effector mechanisms (**Figure 10**).

Genes related to pathogen recognition and activation of the complement system, such as different immune lectins, Galectins, Fucolectins, Techylectin, C1q domain-containing proteins, Ficolins and Fibrinogen-related proteins (FREPs), were upregulated in gills. Galectins interact and recognize different bacterial surface glycans, and their upregulation in *M. galloprovincialis* gills 24 h after *V. splendidus* infection has previously been reported (44). The high variability of FREP sequences in mussels suggests an extraordinarily complex innate immune system in terms of antigen recognition (45). FREP recognition proteins were upregulated in gills but downregulated in the transcriptomic study of hemocytes against a systemic infection (32). C1q domain proteins, involved in antigen and pattern recognition, have also experienced phenomena of gene duplication and expansion in bivalves, presenting high transcript variability with different modulation against the infection (46, 47). The recognition functions of these different complement-related secreted proteins in gills could lead to the activation of inflammatory response and to antibacterial effector mechanisms as consequence of their complement-activation signaling (48, 49). The perlucin-like protein (Aggrecan) is the major proteoglycan component of the extracellular matrix and plays roles in immune recognition by interacting with bacterial surface ligands and with the complement pathway. Aggrecan was strongly modulated with up- and downregulated transcripts. An upregulation of this gene 12 h after Vibrio infection and the respective expression decrease within 24 h had been observed in mussels (44). A transcriptomic study of *M. edulis* hemocytes challenged *in vitro* with *V. splendidus* detected different transcriptomic expression kinetics (50), but the timing of expression regulation *in vitro* may be very different from that in the case of a natural bath infection, and further research needs to be performed on this topic.

Several Toll-like receptor (TLR) genes that can specifically recognize LPS and gram-negative bacterial infections were also upregulated. These molecules are described as leading to the NF-kB-dependent production of pro-inflammatory cytokines and immune gene expression in mussels through signaling pathways that include MyD88 and TRAF among various molecular signaling intermediators, which were also upregulated in our transcriptomic data (49, 51, 52). The expression of these pro-inflammatory cytokines was also detected in gills as it happened with interleukin 17 (IL-17) genes and the allograft inflammatory factor AIF-1. Various IL-17 isoforms were significantly upregulated. These cytokines are implicated in the triggering of the NF-kB inflammatory response in mussel (49, 53). It has also been reported the up-regulation of IL-17 after bacterial infections in other marine invertebrates as oyster and sea urchin (54, 55). IL-17 genes were not modulated in the hemocyte transcriptomic response against a systemic infection (32), being exclusive for the gill response when comparing both mussel transcriptomes. These cytokines could be crucial in the immune triggering of marine invertebrates, linking mucosal immunity recognition and effector functions.

Genes whose expression is activated by the NF-kB signaling pathway were also determined to be among the most upregulated, such as Tetraspanins, Schlafen proteins and Syntenin-1. Tetraspanins play roles in cell adhesion and migration (56), and their upregulation had been observed before in different mollusks upon stimulation with live bacteria in gills and hemocytes (57, 58). Schlafen (Slfn family) proteins are regulators of immune cell development whose transcription is upregulated by TLR signaling under bacterial stimulation (59). The syntenin-1 gene is also expressed by TLR signaling and codes for a multifunctional adaptor protein implicated in the bacterial clearance response, as has been reported in crustaceans (60) and in exosome biogenesis (61). Exosome formation by host or pathogen cells could play opposite roles in bacterial infections (62, 63). More genes related to exosome regulation were found in the gill immune response, such as the CD63 tretraspanin homolog, which was associated with modulated lncRNAs.

The downregulated genes were examined, and the inhibition of some immunosuppressive or anti-inflammatory genes was observed, such as the low-density lipoprotein receptor-related protein LRP1. It was recently described that LRP1 can be modulated by TLR signaling and that it could modulate the immune response. LRP1 inhibition leads to an enhanced sensitivity to lipopolysaccharide (LPS), a stimulus of Gram-negative bacterial infections, activating the production of pro-inflammatory cytokines through NK-kB and JNK pathways (64, 65). In our work, inhibition of this gene was accompanied by a strong response related to cytokines. Two genes implicated in the metabolism of arachidonic acid whose overexpression can cause pathological and autoimmune effects were also downregulated: arachidonate 5-lipoxygenase and its chaperone, coactin-like protein (66–68). Mytimacin and defensin were also downregulated against infection, as has been previously reported with other AMPs under bacterial stimulus (32, 69, 70). AMPs modulation can be related to physiological and stress stimulus as tissular damage (32), which can be derived from the infection. Most likely, mussels do not need to express or produce more AMPs to face the bacteria because they can release the already-synthesized AMPs from the granulocytes more rapidly.

Modulation of the gene expression response was accompanied by a significant modulation of lncRNAs against infection. Little is known regarding lncRNA-mediated gene expression regulation in mollusks, but the evidence of their involvement in the modulation of the response is increasing, including the discovery of regulatory functions over IL-17 expression in oysters (71, 72). In the present work, we report the significant modulation of several lncRNAs against bacterial infection, as well as their potential target genes in the mussel genome. These lncRNAs may model the expression of neighboring protein-encoding genes that have been determined to be primarily involved in the regulation and modulation of immune processes, such as Toll-like receptor precursors and MyD88 proteins, which have been implicated in the NF-kB-mediated response, or the ATP-dependent RNA helicase DDX58 (RIG-I), an innate immune response receptor that leads to pro-inflammatory cytokine production. Additionally, CD63 exosome-related protein, galaxin or TBC1 domain family member 5 were other genes modulated by lncRNAs that could also be implicated in the explained immune responses.

In prior research, hemocytes have been studied as the primary immune effector in the context of mussel infectious diseases, but when comparing the response of these key immune cells and gills, important differences have been found at the transcriptomic level (32). DEGs found only in gills were related to pathogen recognition, adhesion and the pro-inflammatory immune signaling pathways explained previously. These genes were not modulated in internal hemocytes sampled from the muscle, which were more strongly characterized by immune effectors and proteins downstream of those recognition and signaling processes. Shared modulated genes reflected the fact that hemocytes are also infiltrated in gill filaments. These common DEGs were implicated in different functions, such as intracellular pathogen recognition and immune activation, such as the stimulator of interferon genes (STING), which is a sensor of cytosolic DNA from bacteria and viruses, deoxynucleoside triphosphate triphosphohydrolase (SAMHD1) or downregulated C-type hepatic-like lectin, whose overexpression could inhibit the production of pro-inflammatory cytokines (69). Other common DEGs were related to effector functions: interferon-related immune genes, such as IFN-induced protein 44-like and IFN-induced helicase C, or Cathepsin-L, which is involved in protein degradation in lysosomes and is modulated in bivalves in response to infections (73, 74).

Transcriptomic studies in mussel naive gills have already revealed that this tissue is particularly rich in abundant transcripts related to structure and recognition (75). Transcriptomic studies performed in different animals, such as the Atlantic cod *Gadus morhua* or the vent mussel *Bathymodiolus azoricus*, also indicated that gills were particularly rich in the expression of antibacterial and cytokine genes related to immune recognition and signaling (76, 77).

In conclusion, mussels appear to be capable of controlling the entrance of bacterial pathogens in their tissues, both in natural conditions and after an experimental bath infection. In this work, it was confirmed that gills are not only a filtering tissue but also contain components with antibacterial properties. The transcriptomic analysis and the functional antibacterial experiments performed in this study demonstrated for the first time how gills act as a sort of immune recognition tissue in a bath infection, as would happen in the natural environment, modulating the expression of genes responsible for the specific binding of the pathogens and for the activation of the immune response against them. The gill transcriptomic response specializes in the recognition and transduction of signaling processes that activate mussel immune effectors and hemocytes. These findings underscore the importance of focusing not only on hemocytes as the main defensive cells but also on other important tissues, such as gills, to improve our knowledge of the efficient and complex innate immune system of mussels.

## Data Availability Statement

The datasets generated for this study can be obtained in the NCBI Short Read Archive database under the accession IDs SRR11996464, SRR11996686, SRR11996723, SRR11996734, SRR11996735 and SRR11996743, all of which are accessible via the Bioproject ID PRJNA638821.

## Ethics Statement

The Mediterranean mussel, *M. galloprovincialis*, is not considered an endangered or protected species in any international species catalog, including the CITES list (www.cites.org), and it is not included in the list of species regulated by the EC Directive 2010/63/EU. Therefore, no specific authorization is required to work on mussel samples.

## Author Contributions

BN and AF conceived and designed the project. AS and MR-C performed the mussel infection, sampling and RNA extraction. AF and AS performed the bioinformatics analyses. BN and AS analyzed the generated data. AS performed the functional assays. AS wrote the manuscript. All authors contributed to the article and approved the submitted version.

## Funding

This research was funded by the Spanish Ministerio de Ciencia, Innovación y Universidades (AEI/EU-FEDER RTI2018-095997-B-I00) and the EU-H2020 VIVALDI (678589). Our laboratory is funded by Interreg VA Spain-Portugal cooperation programme (POCTEP) 2014-2020, 0474_BLUEBIOLAB project, co-funded by the European Regional Development Fund (FEDER) and IN607B 2019/01 from Consellería de Economía, Emprego e Industria (GAIN), Xunta de Galicia. MR-C was supported by a Spanish AEI/EU-FEDER predoctoral contract BES-2016-076302. AS was supported by a Spanish AEI/EU-FSE predoctoral contract PRE2019-090760.

## Conflict of Interest

The authors declare that the research was conducted in the absence of any commercial or financial relationships that could be construed as a potential conflict of interest.
